# A Prospective Study of the Causes of Febrile Illness Requiring Hospitalization in Children in Cambodia

**DOI:** 10.1371/journal.pone.0060634

**Published:** 2013-04-09

**Authors:** Kheng Chheng, Michael J. Carter, Kate Emary, Ngoun Chanpheaktra, Catrin E. Moore, Nicole Stoesser, Hor Putchhat, Soeng Sona, Sin Reaksmey, Paul Kitsutani, Borann Sar, H. Rogier van Doorn, Nguyen Hanh Uyen, Le Van Tan, Daniel Paris, Stuart D. Blacksell, Premjit Amornchai, Vanaporn Wuthiekanun, Christopher M. Parry, Nicholas P. J. Day, Varun Kumar

**Affiliations:** 1 Angkor Hospital for Children, Siem Reap, Kingdom of Cambodia; 2 Mahidol-Oxford Tropical Medicine Research Unit, Faculty of Tropical Medicine, Mahidol University, Bangkok, Thailand; 3 Centre for Tropical Medicine, Nuffield Department of Clinical Medicine, University of Oxford, Oxford, United Kingdom; 4 Institute of Child Health, University College London, London, United Kingdom; 5 Oxford University Clinical Research Unit, Hospital for Tropical Diseases, Ho Chi Minh City, Vietnam; 6 Influenza Program, US Centers for Disease Control and Prevention, Cambodia Office, Phnom Penh, Cambodia; University of Cambridge, United Kingdom

## Abstract

**Background:**

Febrile illnesses are pre-eminent contributors to morbidity and mortality among children in South-East Asia but the causes are poorly understood. We determined the causes of fever in children hospitalised in Siem Reap province, Cambodia.

**Methods and Findings:**

A one-year prospective study of febrile children admitted to Angkor Hospital for Children, Siem Reap. Demographic, clinical, laboratory and outcome data were comprehensively analysed. Between October 12^th^ 2009 and October 12^th^ 2010 there were 1225 episodes of febrile illness in 1180 children. Median (IQR) age was 2.0 (0.8–6.4) years, with 850 (69%) episodes in children <5 years. Common microbiological diagnoses were dengue virus (16.2%), scrub typhus (7.8%), and Japanese encephalitis virus (5.8%). 76 (6.3%) episodes had culture-proven bloodstream infection, including *Salmonella enterica* serovar Typhi (22 isolates, 1.8%), *Streptococcus pneumoniae* (13, 1.1%), *Escherichia coli* (8, 0.7%), *Haemophilus influenzae* (7, 0.6%), *Staphylococcus aureus* (6, 0.5%) and *Burkholderia pseudomallei* (6, 0.5%). There were 69 deaths (5.6%), including those due to clinically diagnosed pneumonia (19), dengue virus (5), and melioidosis (4). 10 of 69 (14.5%) deaths were associated with culture-proven bloodstream infection in logistic regression analyses (odds ratio for mortality 3.4, 95% CI 1.6–6.9). Antimicrobial resistance was prevalent, particularly in *S. enterica* Typhi, (where 90% of isolates were resistant to ciprofloxacin, and 86% were multi-drug resistant). Comorbid undernutrition was present in 44% of episodes and a major risk factor for acute mortality (OR 2.1, 95% CI 1.1–4.2), as were HIV infection and cardiac disease.

**Conclusion:**

We identified a microbiological cause of fever in almost 50% of episodes in this large study of community-acquired febrile illness in hospitalized children in Cambodia. The range of pathogens, antimicrobial susceptibility, and co-morbidities associated with mortality described will be of use in the development of rational guidelines for infectious disease treatment and control in Cambodia and South-East Asia.

## Introduction

Febrile illness in children is a common cause of admission to hospital globally, with significant associated morbidity and mortality [1]. In developing countries this is frequently compounded by low rates of immunisation, untreated co-morbidities, and late presentations [2]. Febrile illnesses are caused by diverse pathogens, presenting with non-specific symptoms to healthcare facilities with limited diagnostic capacity [3], [4]. Clinical management guidelines for acute febrile illness are available [5], [6], but rarely supported by knowledge of the locally prevalent causative agents.

The Kingdom of Cambodia lies in South-East Asia and has a mortality rate in children aged <5 years of 54/1000 live births [7]. This has halved over the last decade but remains one of the highest in the region. The prevalence of undernutrition in children <5 years of age (less than 2 SD of weight for age) is 28% [7]. There is little published information on the causes of fever in Cambodian children.

We characterised the causes of febrile illness in children in Cambodia. We hypothesised that in addition to globally common childhood pathogens such as *Streptococcus pneumoniae* and influenza virus, other infections that require specific management would be identified, such as typhoid, dengue, leptospirosis, melioidosis and rickettsial disease [4], [8]–[19].

## Materials and Methods

### Ethics Statement

Parents of all children recruited to the study gave witnessed, written, informed consent before study enrolment. The Oxford Tropical Research Ethics Committee and Angkor Hospital for Children Institutional Review Board approved the study protocol on 24th September 2009 and 2nd October 2009 respectively.

### Study Site and Population

**Figure 1:**
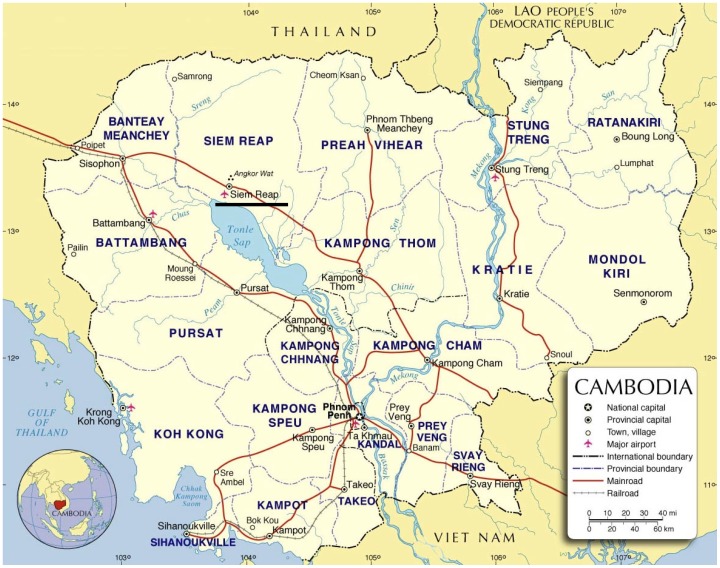
Location and map of Cambodia. AHC is in Siem Reap (underlined). Map: United Nations, 2004 (http://www.un.org/Depts/Cartographic/map/profile/cambodia.pdf). Accessed 2nd January 2013.

This prospective, year-long study of the causes of fever in children was based at Angkor Hospital for Children (AHC), Siem Reap province, Cambodia (**Figure 1**). AHC is a 50-bed paediatric hospital providing free universal inpatient and outpatient care to children <16 years of age from urban and rural settings. It has critical care capacity, including mechanical ventilation and inotropic support, and is one of two paediatric hospitals serving Siem Reap city and province.

The national immunisation schedule included Bacillus Calmette-Guérin (BCG) and hepatitis B virus (HBV) at birth, and diphtheria-pertussis-tetanus, oral poliovirus and measles virus vaccines. 79% of children aged 12–23 months have received these vaccines [7]. *Haemophilus influenzae* type b immunisation was introduced during the study period.

### Patients and Clinical Methods

Patients admitted to AHC between 12^th^ October 2009 and 12^th^ October 2010 were considered for enrolment. Eligibility criteria were age <16 years, documented axillary temperature ≥38.0°C within 48 h of admission, and caregiver consent. Febrile post-surgical patients were excluded. All children, except emergencies, were assessed using locally-modified *Integrated Management of Childhood Illness* (*IMCI*) guidelines [5] prior to a decision to admit. Admission information was recorded on a study-specific clinical record form. Admissions were reviewed twice daily for eligibility.

### Sampling and Laboratory Methods

Blood was taken aseptically from all enrolled patients for culture, complete blood count, blood film (including malaria smear), biochemistry, and nucleic acid amplification tests (NAATs). In addition, study patients aged ≥60 days had admission blood samples taken for serology and *Leptospira* spp. culture, and a convalescent serology sample taken on discharge, or after 7 days of admission. Whole blood and serum samples were stored at –80°C until analysed.

Nasal and throat swabs were taken for respiratory virus detection in patients with a recent history of cough or sore throat, and increased respiratory effort. Cerebrospinal fluid (CSF) analysis was performed on patients with suspected central nervous system (CNS) infection. Urinalysis was performed on all children; other sampling (e.g. HIV serology, gastric aspirates) and imaging were performed as clinically indicated.

### Bacterial Culture

Blood was taken for culture, when possible before in-hospital antimicrobial therapy and within 48 h of admission. Blood was inoculated into a pre-weighed blood culture bottle and the vented bottles were incubated aerobically at 37°C for 7 days [20]. Sub-culture onto sheep blood and chocolate agar was undertaken at 24 h, 48 h, 7 days, or if the culture was turbid. CSF samples were separated immediately upon receipt into aliquots for staining and microscopy, cell count and biochemistry, culture, and storage at –80°C. Cultured organisms were identified using routine methods [20], including API test kits (bioMérieux, France), disc diffusion antimicrobial susceptibility testing [21] and Etests™ (AB Biodisk, Sweden) performed as appropriate. Samples from other sterile sites (e.g. abscesses, pleural fluid) were cultured using routine methods [20] and were reviewed daily for clinical relevance by the infectious diseases team. Whole blood samples were cultured for *Leptospira* spp. [22].

### Serology

The Panbio Japanese encephalitis virus (JEV) and dengue virus (DENV) IgM Combo enzyme-linked immunosorbent assay (ELISA) (Panbio, Australia) was used to detect anti-JEV and anti-DENV specific IgM antibodies in sera. An ELISA (Standard Diagnostics, Korea) was used to detect DENV NS1 antigen [23]. A capture IgM ELISA assay (Venture Technologies, Malaysia) was used to detect anti-JEV and anti-DENV specific IgM antibodies in CSF specimens [24]. Table S1 describes the result interpretation.

Separate ELISAs incorporating *Orientia tsutsugamushi* (Karp and Gilliam strain) and *Rickettsia typhi* (Wilmington strain) antigens were used to detect *O. tsutsugamushi* and *R. typhi* IgM antibodies in serum samples respectively [25]. A positive ELISA result was equivalent to a (conservative) 1∶200–1∶400 indirect immunofluorescence assay (IFA) titre [26] (SD Blacksell: unpublished data). “Acute positive serology” was diagnosed in paired samples with a ≥4-fold increase in IFA IgM antibody titres. Serum pairs with <4-fold increase in IFA IgM antibody titres, or a single sample with a titre ≥1∶400, were recorded as “acute/recent positive serology” [25].

### Nucleic Acid Amplification Tests

DNA was extracted from whole blood samples with the QIAamp® DNA mini-kit (QIAGEN, Germany), with extended incubation for 30 minutes at 56°C. Probe-based real-time polymerase chain reaction (rPCR) assays were used to detect *Leptospira* spp. [27], *O. tsutsugamushi* [28] and *R. typhi* [29]. Low-positive plasmid or sample controls determined adequate detection limits of each assay.

Total nucleic acid was isolated from 100 µL of CSF specimens using the automated easyMAG® system (bioMérieux), and diagnostic NAATs were performed [30]. Four rPCR protocols were used for detection of *S. pneumoniae*, *H. influenzae* type B, *Neisseria meningitidis*, and *Streptococcus suis* [30]. rRT-PCRs were used to detect herpes simplex virus (HSV) 1 and 2 [31], varicella zoster virus (VZV) [32], enteroviruses (generic and 71-specific) [33], and human parechoviruses (generic) [34].

Nasal and throat swabs were stored in viral transport medium (Becton Dickinson, USA) at 4°C for ≤72 h, then divided and stored at –80°C. RNA was extracted from one aliquot of the pooled swab medium using the QIAamp® Viral RNA mini-kit (QIAGEN). Specimens were tested for influenza virus (types A, B; and subtypes H1N1-1977, H1N1-pdm09, H3N2 and H5N1) by real-time reverse-transcription PCR (rRT-PCR), and for RSV, parainfluenza virus (PIV)1, PIV2 and PIV3 using multiplex RT-PCR (QIAGEN) [35]–[38].

Full details are in the supplementary material. All nucleic acid extractions and assays were done according to the manufacturer’s instructions.

### Clinical Data

For each episode, the first recorded parameter after the time of admission was used. The clinical syndrome was categorised by a senior paediatrician (VK) at hospital discharge according to the localising focus of infection, e.g. lower respiratory tract infection (LRTI), or non-infectious cause for fever. On final analysis of all data, a microbiological diagnosis was given when results were consistent with the presenting clinical syndrome. When microbiological results were inconsistent with the clinical syndrome, both were given as a final diagnosis. Two clinicians (KE and MC) made these judgements independently with disagreements resolved by discussion.

### Data Management and Analysis

Data was managed on a study-specific database and analysed using Stata 12 (Stata Corp., USA), with weight-for-age z-scores calculated using the WHO Anthro 3.2.2 Stata macro (World Health Organisation). Multivariate logistic regression was used to analyse the effects of comorbid undernutrition, heart disease, HIV infection, and anaemia; adjusted for each other and age group.

## Results

### Baseline Characteristics

**Figure 2:**
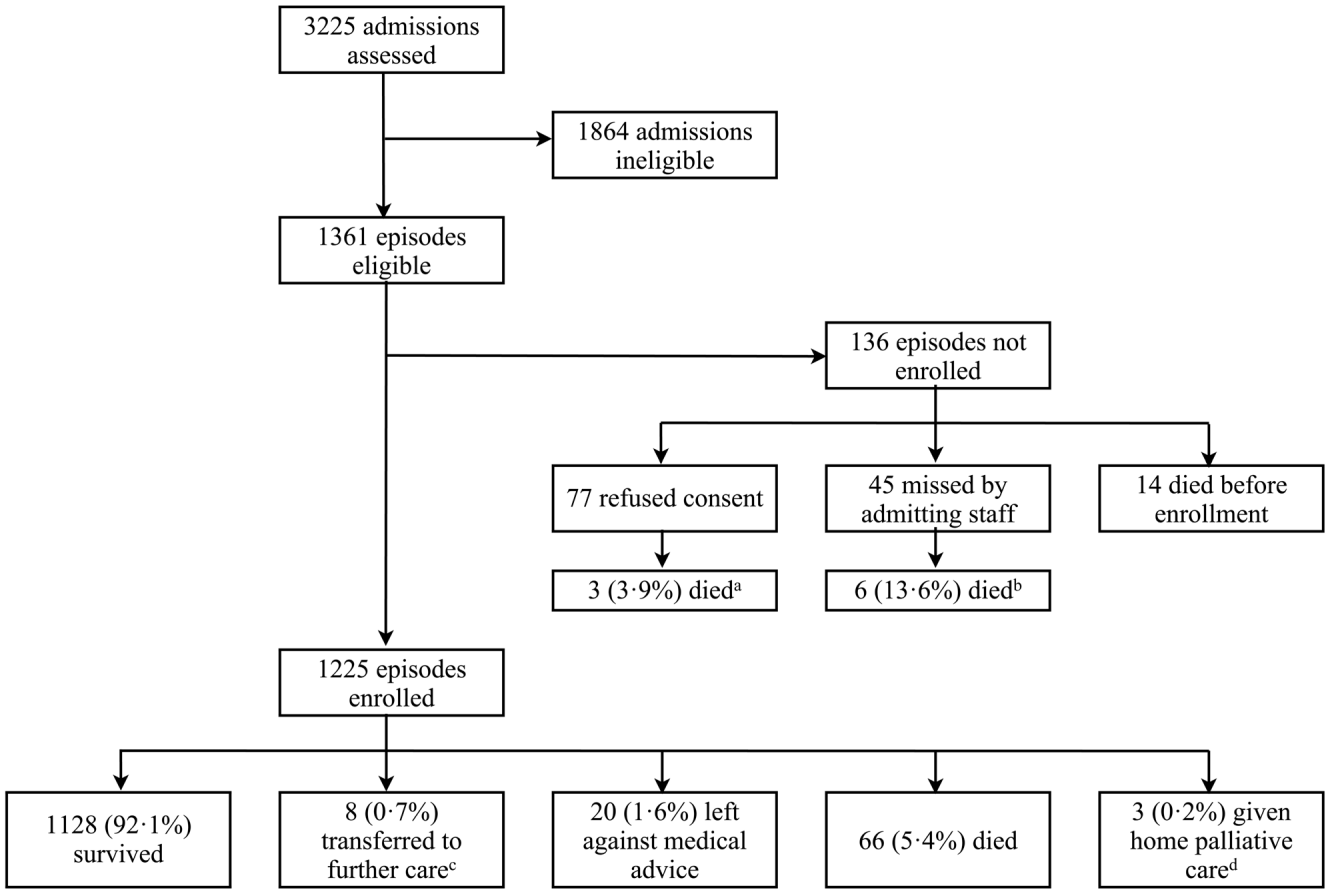
Flow chart showing enrolment to the study. Notes: ^a^including one home palliative care; ^b^including one home palliative care; ^c^excluded from analyses of outcome (e.g. in odds ratios); ^d^included as “died” in analyses.

There were 3225 patient admissions during the study year, of which 1361 (42.2%) met the inclusion criteria. Of these, 136 (10.0%) were not enrolled, leaving 1225 febrile episodes in 1180 children (**Figure 2**). 1144 children had a single episode, 31 children had two episodes, one child had three episodes and four children had four episodes. The median (inter quartile range [IQR]) age was 2.0 (0.8–6.4) years, with 850 (69.4%) episodes in children <5 years of age. The median (IQR) duration of illness prior to admission was 3 (2–5) days. Medication was given by the caregiver prior to admission in 53% of episodes. In 43% of these the medication was a known antibacterial, anti-malarial or steroid. Other baseline characteristics are in **Table 1**.

**Table 1:** Baseline, comorbidity and outcome characteristics in 1225 disease episodes among 1180 children admitted to hospital with fever over one year.

Characteristic	Number or median (IQR^a^, range)
**Demographics**	
Number of episodes	1225
Male	668 (54.5%)
Median (IQR, range) age (years)	2.0 (0.8–6.4, 0.0–16.0)
Numbers in each age range:	
<28 days	32 (2.6%)
28 days to <1 year	330 (26.9%)
≥1 year to <5 years	488 (39.8%)
≥5 years to <16 years	375 (30.6%)
**Origin**	
Acute transfer from other healthcare facility	161 (13.2%)
Residence in Siem Reap district	269 (22.0%)
Residence in Siem Reap province (not district)	542 (44.2%)
Residence in neighbouring provinces	371 (30.3%)
Residence in distant provinces	43 (3.5%)
**Known comorbidity** b	
HIV infection	58 (4.7%)
Hepatitis B infection	5 (0.4%)
Hepatitis C infection	1 (<0.1%)
Mean weight-for-age z-score (95% CI)	–1.97 (–4.5–+0.3)
Undernutrition (weight-for-age z-score <2 standard deviations below mean in those <5 yrs of age)	371 (43.4%)
Congenital or rheumatic heart disease (on echocardiograpy)	81 (6.6%)
Severe anaemia (<7.0 g/dL) on admission	99 (8.1%)
**Inpatient episode**	
Median (IQR) duration of fever before hospitalisation (days)	3 (2–5)^c^
Median (IQR, range) duration of hospitalisation (days)	4 (3–8, 0–123)
Admitted to critical care unit at least once during admission	300 (24.5%)
Requiring respiratory support	192 (15.7%)
Needing CPAP alone	81 (7.3%)
Needing mechanical ventilation	111 (9.1%)
**Mortality**	
Died or discharged to die at home	69 (5.6%)
Median (IQR, range) time to death (n = 69) (days)	4 (2–10, 0–123)

a IQR interquartile range.

b *n* = 1180 children except the z-score which was generated for those children <5 years of age using WHO (2006) data where *n* = 855.

c 3 children had caregiver reported fever for >1 month.

### Clinical Syndrome Diagnoses

A total of 1333 presenting clinical syndromes were noted in 1225 febrile episodes, with the most common being LRTI (38.3% of episodes), undifferentiated fever (25.5%) and diarrhoeal disease (19.5%) (**Table 2**). 575 (46.9%) episodes were positive for a microbiological cause of fever results by any method (culture, NAAT, direct stain, serology), with 731 microbiological causes of fever diagnosed within these 575 episodes (**Table 3**). A consistent microbiological diagnosis was made in 125 of 472 (26.5%) episodes of LRTI, 77 of 267 (28.8%) episodes of diarrhoeal disease, and 66 of 148 (44.6%) episodes of undifferentiated fever.

**Table 2:** The presenting clinical syndromes diagnosed for 1225 episodes of febrile illness over the one-year study period.

	Age groups				
Clinical syndrome	<28 days (*n* = 32)	28–365 days (*n* = 330)	1–5 years (*n* = 488)	≥5 years (*n* = 375)	Total (*n* = 1225)
Lower respiratory tract infection	8 (25.0%)	183 (55.5%)	218 (44.7%)	60 (16.0%)	469 (38.3%)
Undifferentiated fever	13 (40.1%)	36 (10.9%)	98 (20.1%)	165 (44.0%)	312 (25.5%)
Diarrhoeal disease	4 (12.5%)	104 (31.5%)	110 (22.5%)	21 (5.6%)	239 (19.5%)
Skin/soft tissue/bone/joint infection	4 (12.5%)	10 (3.0%)	28 (5.7%)	46 (12.3%)	88 (7.2%)
Upper respiratory tract infection	0	14 (4.2%)	36 (7.4%)	14 (3.7%)	64 (5.2%)
CNS infection	1 (3.1%)	12 (3.6%)	10 (2.0%)	33 (8.8%)	56 (4.6%)
Genitourinary	0	14 (4.2%)	12 (2.4%)	15 (4.0%)	41 (3.4%)
Abdominal disease/surgical abdomen	0	0	3 (0.6%)	18 (4.8%)	21 (1.7%)
Non-infectious cause of fever	2 (6.3%)	6 (1.8%)	16 (3.3%)	19 (5.1%)	43 (3.5%)
Total clinical diagnoses					1333
Acute mortality	6 (18.8%)	23 (7.0%)	18 (3.7%)	22 (5.9%)	69 (5.6%)

Within these 1225 episodes, a total of 1333 clinical syndromes were diagnosed: 1120 episodes had a single clinical syndrome diagnosed, 102 episodes had two separate syndromes diagnosed, and three episodes had three separate syndromes diagnosed (i.e. 1333 syndromes in total).

**Table 3:** Microbiological diagnoses.

	Age groups					
	<28 days	28 days–<1 year		≥1–<5 years	≥5–<16 years	Total number
Number of children	32	330		488	375	1225^c^
**Gram-positive bacteria**						
*Staphylococcus aureus*	3	3		11	20	37 (3.0%)
*Streptococcus pneumoniae*	0	2		8	8	18 (1.5%)
*Streptococcus pyogenes*	1	1		1	1	4 (0.3%)
**Gram-negative bacteria**						
*Salmonella enterica* serovar Typhi	0	1		6	15	22 (1.8%)
*Burkholderia pseudomallei*	0	1		6	7	14 (1.1%)
*Escherichia coli*	1	4		5	3	13 (1.1%)
*Haemophilus influenzae*	0	5		4	0	9 (0.7%)
*Klebsiella pneumoniae*	1	3		1	1	6 (0.5%)
*Neisseria meningitides*	0	2		0	2	4 (0.3%)
*Acinetobacter baumannii*	1	1		1	0	3 (0.2%)
*Pseudomonas aeruginosa*	1	1		0	1	3 (0.2%)
Non-typhoid *Salmonella* spp.	0	0		0	1	1 (<0.1%)
*Pantoea* spp.	1	0		0	0	1 (<0.1%)
Blood isolate of uncertain significance	3	9		9	5	26 (2.1%)
Cultured bacterial pathogens (blood, pus and CSF) excluding blood isolates of uncertain significance						135 (11.0%)
**Rickettsioses and other bacteria**						
*Orientia tsutsugamushi*	0	12	21		63	96 (7.8%)
*Rickettsia typhi*	0	4	8		15	27 (2.2%)
*Rickettsia* of indeterminate species	0	0	5		6	11 (0.9%)
**Viruses**						
Dengue virus	0	54	63		81	198 (16.2%)
Japanese encephalitis virus	0	29	27		15	71 (5.8%)
Indeterminate flavivirus	0	22	26		17	65 (5.3%)
Parainfluenza virus 3	0	9	8		1	18 (1.5%)
Respiratory syncytial virus	0	6	7		1	14 (1.1%)
Influenza virus A/H1N1-pdm09	0	3	5		3	12 (1.0%)
Parainfluenza virus 1	1	3	5		1	10 (0.7%)
Enteroviruses (non-71)	0	1	1		6	8 (0.7%)
Influenza virus A/H3N2	0	0	5		2	7 (0.6%)
Influenza virus B	0	3	2		0	5 (0.4%)
Parainfluenza virus 2	0	1	1		0	2 (0.2%)
Influenza virus A/H1N1-1977	0	0	0		1	1 (<0.1%)
Herpes simplex virus	0	0	1		0	1 (<0.1%)
**Malaria**						
*Plasmodium falciparum* only	0	2	6		6	14 (1.1%)
*P vivax* only	0	2	3		2	7 (0.6%)
*P vivax* and *P falciparum*	0	0	0		3	3 (0.2%)
**Other**						
*Leptospira* spp	0	3	8		6	17 (1.4%)
*Mycobacterium tuberculosis* a	0	0	1		5	6 (0.5%)
Yeast (non-cryptococcal)	0	1	0		1	2 (0.2%)
*Cryptococcus neoformans*	0	0	0		1	1 (<0.1%)
**Non-infectious cause of fever** b						17 (1.4%)

a Ziehl-Neelsen stain only, no culture facilities available.

b Non-infectious causes of fever included 11 episodes attributed to haematological malignancy, five episodes attributed to burns and one episode attributed to juvenile idiopathic arthritis.

c Using *n* for entire cohort, see text and **Tables 4** **–** [Table pone-0060634-t005] [Table pone-0060634-t006] [Table pone-0060634-t007] **8** for denominators by each test.

### Blood and Sterile Site Sampling

Blood was cultured within 48 h of admission in 1212 (99.0%) episodes. The mean volume of blood drawn was 1.7 mL (95% confidence interval [CI] 1.0–3.3) for children aged <28 days, and 2.0 mL (95% CI 1.0–4.0) for those ≥60 days of age. The blood culture was positive in 162 (13.4%) samples, of which 76 (6.3%) were considered true positives, 26 (2.1%) were isolates of uncertain significance (**Table 4**), and 60 (5.0%) considered contaminants. 10 of 69 (14.5%) deaths were associated with a true positive blood culture (Odds Ratio [OR] for mortality 3.4, 95% CI 1.6–6.9). 5 of 69 (7.2%) deaths were associated with isolates from blood culture of uncertain significance (OR adjusted for age 3.8, 95% CI 1.4–10.4). Contaminants from blood culture were not associated with mortality (OR adjusted for age 0.8, 95% CI 0.2–2.6). Pus was sampled in 127 (10.8%) of episodes, with the most numerous isolates being *S. aureus* (31) and *B. pseudomallei* (8).

**Table 4:** Isolates from blood cultures (*n* = 1212).

Isolate from blood culture	Number of positive cultures
*S. enterica* Typhi	22^a^ (1.8%)
*S. pneumoniae*	13^b^ (1.1%)
*E. coli*	8^c^ (0.7%)
*H. influenzae*	7 (0.6%)
*B. pseudomallei*	6^e^ (0.5%)
*S. aureus*	6^d^ (0.5%)
*K. pneumoniae*	4^f^ (0.3%)
*Acinetobacter baumannii*	3 (0.2%)
*N. meningitidis*	2 (0.2%)
*S. pyogenes*	2 (0.2%)
Yeast	1 (<0.1%)
*Pantoea* spp.	1 (<0.1%)
*P. aeruginosa*	1 (<0.1%)
Total true isolates	76 (6.3%)
**Isolates of uncertain significance**	
Unidentified Gram-negative bacilli	5 (0.4%)
*Pseudomonas* spp.	5 (0.4%)
*Acinetobacter calcoaceticus*	4 (0.3%)
*Ochrobactum anthropi*	3 (0.2%)
*Burkholderia cepacia*	2 (0.2%)
*Moraxella* spp.	2 (0.2%)
*Alcaligenes faecalis*	1 (0.1%)
*Gemella morbillorum*	1 (0.1%)
*Sphingobacterium spiritivorum*	1 (0.1%)
*Stenotrophomonas maltophilia*	1 (0.1%)
*Streptococcus sanguinis*	1 (0.1%)
Total isolates of uncertain significance	26 (2.1%)
**Contaminants**	60^g^ (5.0%)

a 19/21 (90.5%) had intermediate susceptibility to ciprofloxacin; 18/21 (85.7%) were multi-drug resistant (resistant to chloramphenicol, ampicillin and co-trimoxozole).

b 8 strains were available for testing, all were susceptible to ceftriaxone.

c 4 strains were available for testing, one was ESBL producing.

d 6 strains available for testing, one was MRSA.

e Constitutively resistant to ceftriaxone.

f 2 strains available for testing, one was ESBL producing.

g 33 coagulase-negative staphylococci, 27 Gram-positive bacilli.

### CSF Sampling

A CSF sample was examined by microscopy and culture from children in 174 (14.2%) of disease episodes. 52 (30.0%) CSF samples showed white cell pleocytosis, and 11 (6.3%) were culture or CSF stain positive. There was sufficient CSF for NAATs and serology from 107 (62.6%) samples, with 15 (14.0%) positive by NAAT and 7 (6.5%) positive by serology (**Table 5**). All CSF samples positive by culture for were also positive by NAATs. NAATs also identified an additional three *S. pneumoniae* and one *H. influenzae* positive episodes.

**Table 5 pone-0060634-t005:** CSF microscopy and culture (*n* = 174), NAAT (*n* = 107) results, and total episodes positive (*n* = 174) by all methods for children with suspected CNS infection.

CSF isolate	Culture positive samples	NAAT positive samples	Serology positive samples	Episodes positive
*H. influenzae*	4 (2.3%)	1 (1.9%)	–	5 (2.9%)
*S. pneumoniae*	2 (1.1%)	4 (3.7%)	–	5 (2.9%)
*N. meningitides*	3 (1.7%)	1 (0.9%)	–	3 (1.7%)
*P. aeruginosa*	1 (0.6%)	–	–	1 (0.5%)
*C. neoformans*	1 (0.6%)	–	–	1 (0.5%)
Enterovirus	–	8 (7.5%)	–	8 (4.6%)
Herpes simplex virus	–	1 (0.9%)	–	1 (0.5%)
Japanese encephalitis virus	–	–	6 (5.6%)	6 (3.4%)
Dengue virus	–	–	1 (0.9%)	1 (0.5%)
Total CSF isolates	11 (6.3%)	15 (14.0%)	7 (6.5%)	31 (19.5%)

### *Leptospira* spp. and Rickettsial Disease

*Leptospira* spp. culture was performed for 1068 of 1149 (93.0%) disease episodes in children ≥60 days of age, and two (0.2%) were positive. NAAT for *Leptospira* spp. was performed in 1179 episodes (96.2%) of all ages and 17 (1.4%) were positive. Both episodes positive by culture were positive by NAAT (**Table 6**).

**Table 6 pone-0060634-t006:** Positive results by culture (*n* = 1068) and NAAT (*n* = 1179) for *Leptospira* spp infections; by serology (*n* = 1125) and NAAT (*n* = 1179) for rickettsial infections; and total episodes positive for *Leptospira* spp and rickettsial infections.

Organism	Culture positive	NAAT positive	Episodes positive
*Leptospira* spp.	2 (0.2%)	17 (1.4%)	17
	**Serology positive**	**NAAT positive**	
Acute *O. tsutsugamushi*	5 (0.4%)	17^a^ (1.4%)	96
Acute/recent *O. tsutsugamushi*	90 (8.0%)		
Acute *R. typhi*	3 (0.3%)	2^b^ (0.2%)	27
Acute/recent *R. typhi*	22 (2.0%)		
Acute/recent *Rickettsia* of indeterminate species	11 (1.0%)	–	11
Total rickettsial infections			131^c^

“Acute rickettsial serology” denotes a >4-fold dynamic rise in specific IgM between acute and convalescent (7-days) samples; “acute/recent serology” denotes either static IgM titres or raised IgM in a single acute sample.

a One additional to serological testing.

b Two additional to serological testing.

c Three additional to serological testing.

Serology for *O. tsutsugamushi* and *R. typhi* was performed in 1125 (98.0%) disease episodes in children ≥60 days of age, and NAATs for *O. tsutsugamushi* and *R. typhi* were done in 1179 (96.3%) of episodes of all ages (**Table 6**). 95 (8.4%) samples were seropositive for anti-*O. tsutsugamushi* IgM. 17 (1.4%) samples were positive by NAAT for *O. tsutsugamushi*, of which 16 were also IgM seropositive. 25 (2.2%) samples were seropositive for anti-*R. typhi* IgM. 2 (0.2%) samples were positive by NAAT for *R. typhi*, neither of which were IgM seropositive. Seropositivity for anti-*O. tsutsugamushi* IgM was negatively associated with seropositivity for anti-*R. typhi* IgM (4.7% for *O. tsutsugamushi* alone versus 4.0% for both; McNemar’s test, p = 0.0005).

### Virology

Serological evidence of a flavivirus infection was determined in 1125 (98.0%) disease episodes in children ≥60 days of age (**Table 7**); DENV NS1 antigen was assayed in 1105 (96.4%) of episodes in children ≥60 days of age and 60 (5.4%) samples were positive. There was pronounced seasonality in “acute serology” to DENV (NS1 antigen positive, dynamic rise in anti-DENV IgM titres, or CSF anti-DENV IgM positive) in contrast to lack of seasonality in “acute/recent serology” to DENV or other flaviviruses (**Figure S2** and **Figure S3**).

**Table 7 pone-0060634-t007:** Serological results for flaviviruses for episodes in children of 60 days or older (*n* = 1125).

Serological interpretation	NS1 positive	NS1 negative	Number of positive samples
Acute dengue virus infection	59	55 ^0^	114 (10.1%)
Acute/recent dengue virus infection	0	84 ^4^	84 (7.5%)
Total dengue virus infections			198 (17.6%)
Acute JEV	1^a^	37 ^1^	38 (3.4%)
Acute/recent JEV	0	34 ^1^	34 (3.0%)
Total JEV infections			72 (6.4%)
Acute indeterminate flavivirus infection	0	0	0
Acute/recent indeterminate flavivirus infection	0	65 ^2^	65 (5.8%)
Total indeterminate flavivirus infections			65 (5.8%)
Total positive to flaviviruses	60	275 ^8^	324 (28.8%)
Negative to flaviviruses	0	801 ^23^	-

By definition, all NS1 positive samples were denoted “acute dengue virus serology”, and all CSF IgM positive samples were “acute serology” (although we acknowledge that NS1 antigen assay is not a serological test). Numbers in superscript denote samples with insufficient serum for NS1 antigen assay.

a CSF positive for anti-JEV IgM, serum positive for NS1 and anti-DENV IgM in same episode (diagnosed with both).

There were 389 febrile episodes that met our criteria to have oral and nasopharyngeal swabs sent for the analysis of respiratory viruses (**Table 8**).

**Table 8:** Positive results by NAAT for respiratory viruses in children presenting with sore throat or cough and increased respiratory rate or effort (*n* = 389).

Respiratory virus	NAAT positive
Parainfluenza virus 3	18 (4.6%)
Respiratory syncytial virus	14 (3.6%)
Influenza virus A/H1N1-pdm09	11 (2.8%)
Parainfluenza virus 1	10 (2.6%)
Influenza virus A/H3N2	7 (1.8%)
Influenza virus B	5 (1.3%)
Parainfluenza virus 2	2 (0.5%)
Influenza virus A/H1N1-1977	1 (0.3%)
Total	68 (17.5%)

### Diagnostic Methods and Multiple Positivity

144 (20.0%) of 731 microbiological causes of fever were diagnosed from direct culture (76 from blood), 440 (60.3%) from serological testing, 115 (15.7%) from NAATs, and 32 (4.4%) from direct stain of CSF, pus or gastric aspirates (for *M tuberculosis*). Of 440 IgM seropositive tests for flaviviruses and rickettsias, 157 (35.7%) showed clear “acute serology”, with the remainder showing “acute/recent serology” (as defined above). Median time between acute and discharge/convalescent samples was 4 days (IQR 2–7).

444 (77.2%) of 575 microbiologically positive episodes had one microbiological cause of fever, 108 (18.8%) had two microbiological causes, 21 (3.7%) had three microbiological causes, and 2 (0.3%) had four microbiological causes of fever (excluding bacteria of uncertain significance from blood culture).

10 (5.1%) episodes with anti-DENV IgM seropositivity were also positive for invasive bacterial disease from blood culture (7 with *S. enterica* Typhi, 1 each with *H. influenzae*, *K. pneumoniae* and *S. aureus*). DENV infection was positively associated with malaria (McNemar’s test, p<0.0001) with 11 (5.6%) episodes seropositive for anti-DENV IgM also malaria positive (6 of which *P. falciparum* positive).

### Mortality

During the one-year study, 69 (5.6%) children died during their acute illness (**Table 9**). Common associations (**Table 1** for definitions and prevalence) with mortality in our cohort (adjusted for comorbid undernutrition, HIV infection, and heart disease), were comorbid undernutrition in children <5 years of age (OR 2.1 [95% CI 1.1–4.2]; population attributable fraction [PAF] = 0.31 [95% CI 0.10–0.52]), comorbid heart disease (OR = 3.3 [95% CI 1.5–6.9]; PAF = 0.14 [95% CI 0.02–0.25]), comorbid HIV infection (OR = 3.9 [95% CI 1.2–12.8]; PAF = 0.06 [–0.02–0.13]). 68 of 69 (98.9%) children who died were admitted to the critical care unit.

**Table 9:** Primary diagnosis, and contributing diagnoses, for the 69 children who died during the study.

Primary diagnosis	Contributing diagnoses	Number of deaths(*n* = 69)
**Invasive organisms**		
*B. pseudomallei* bacteraemia	DENV infection (1), *O. tsutsugamushi* (1)	4 (5.8%)
*E. coli* bacteraemia	–	3 (4.3%)
*S. pneumoniae* meningitis	*O. tsutsugamushi* (1)	2 (2.9%)
*S. aureus* pyomyositis/arthritis	–	2 (2.9%)
*Acinetobacter* spp bacteraemia	HIV (1)	2 (2.9%)
*H. influenzae* bacteraemia	–	1 (1.4%)
*K. pneumoniae* bacteraemia	–	1 (1.4%)
*Pantoea* spp (surgical case)	–	1 (1.4%)
*M. tuberculosis* pneumonia	HIV	1 (1.4%)
*C. neoformans* meningitis	HIV	1 (1.4%)
*O. tsutsugamushi* rickettsaemia	DENV infection	1 (1.4%)
Total invasive organisms		19 (27.5%)
**Viral infections**		
DENV infection	*O. tsutsugamushi* (1)	5 (7.2%)
Influenza virus A/H1N1-pdm09	–	2 (2.9%)
Parainfluenza 1 bronchiolitis	*S. aureus* cellulitis (1), *O. tsutsugamushi* and uncertain flavivirus (1)^a^	2 (2.9%)
JEV infection	Tetralogy of Fallot	1 (1.4%)
RSV bronchiolitis	–	1 (1.4%)
Total viral infections		11 (15.9%)
**Primary clinical diagnoses**		
Clinical pneumonia	–b	12 (17.4%)
	DENV infection	4 (5.8%)
	HIV	2 (2.9%)
	Clinical diarrhoea	1 (1.4%)
Unknown source of fever	–	11 (15.9%)
	Leukaemia	2 (2.9%)
Clinical diarrhoea	–c	1 (1.4%)
	Clinical TB, HIV	1 (1.4%)
	HIV^d^	1 (1.4%)
Clinical CNS infection	–d	1 (1.4%)
Clinical tetanus (neonatal)	–	1 (1.4%)
Clinical UTI	–	1 (1.4%)
Clinical parotitis	–	1 (1.4%)
Total primary clinical diagnoses	–	39 (56.5%)

These diagnoses are based on all the available results: hence are not identical to “clinical syndrome diagnosed” in **Table 2**. Numbers in parentheses indicate the number of patients with the contributing diagnosis in addition to the primary diagnosis. Superscripts give further information about individual morbid patients:

a Also with organism of uncertain significance from blood (*Burkholderia cepacia*) and pulmonary hypertension secondary to congenital heart disease;

b also with organism of uncertain significance from blood (*Acinetobacter calcoaceticus*);

c also with organism of uncertain significance from blood (*A. calcoaceticus*);

d also with organism of uncertain significance from blood (*Ochrobactrum anthropi*);

e also with organism of uncertain significance from blood (unidentified Gram-negative bacilli).

Importantly, 23 children of 136 eligible but non-enrolled episodes died (16.9%), and their microbiological data are therefore unavailable (**Figure 2**). 10 of 19 enrolled children (52.6%) who died with a primary diagnosis of “clinical pneumonia” (**Table 9**) had samples for respiratory viruses sent for analysis; and 1 of 11 enrolled children (9.1%) who died with a primary diagnosis of “unknown source of fever” (**Table 9**) had CSF samples analysed.

## Discussion

With comprehensive laboratory investigation we identified a microbiological cause in almost 50% of febrile illness in this one-year study of febrile Cambodian children requiring admission to hospital. The acute mortality rate was 5.6% of children enrolled (6.7% of all eligible). Nurse-led triage using *IMCI* [5] guidelines and paediatric review prior to admission excluded less-severe infections. Children died despite availability of parenteral broad-spectrum antimicrobials, critical care, and laboratory facilities, all unavailable to the majority of the Cambodian population. Prevalence of comorbid undernutrition was 43.7%, and doubled the odds of mortality. Comorbid heart disease, and known HIV infection were also associated with large increases in odds of mortality.

LRTIs, diarrhoeal disease or undifferentiated fever were the main presentations, but a microbiological diagnosis was achieved in only 27%, 29%, and 45% of episodes with these syndromes, respectively. The absence of microbiological examination of faeces, limited use of urine culture, and lack of *M. tuberculosis* culture (or NAAT) facilities were limitations. More inclusive criteria for the analysis of respiratory viruses, HIV testing, and greater emphasis on CSF sampling may have increased our diagnostic yield. In contrast to a recent report [33], we found no enterovirus-71 in 8 episodes of CSF enterovirus-positive meningoencephalitis.

The serological data from our study is consistent with similar studies in Cambodia [8], [14], and neighbouring urban Laos [10]. There was evidence of DENV infection in 16% of episodes, but clinical differentiation from bacterial septic shock was difficult, with children frequently treated for both. This strategy is supported by the co-incidence of both invasive bacterial disease (particularly *S. enterica* Typhi) and malaria, with DENV infection in our cohort. Serological evidence of infection by *O. tsutsugamushi* and *R. typhi* was also common. Although the interpretation of serological tests on cohorts of unselected febrile children can be difficult [25], [26] even with conservative cut-offs for IgM titres against *O. tsutsugamushi* and *R. typhi*, we estimated that 10% of children were infected with these microorganisms. NAATs may increase the specificity of diagnosis, but may lack sensitivity due to short periods of rickettsaemia.

Invasive community-acquired bacterial disease was common, frequently resistant to commonly used antimicrobials, and associated with significant mortality. The most common isolate from blood was *S. enterica* Typhi, with 90% of isolates with intermediate resistance to ciprofloxacin, and 85% multi-drug resistant (MDR) [18], contrasting with the decline in drug-resistant phenotypes seen in adjacent countries [12]. *B. pseudomallei* (inherently resistant to ceftriaxone and penicillins) was confirmed as a pathogen of major local public health significance [16], and *E. coli* (of which 1 of 7 isolates demonstrated extended-spectrum β-lactamase activity) was also prevalent. *S. aureus* was the commonest isolate from all sterile sites, with one meticillin-resistant isolate [19]. Invasive disease caused by *S. pneumoniae*, *H. influenzae*, and *N. meningitidis* is present and potentially vaccine-preventable. The small numbers of neonates in this cohort (not due to refusal of parental consent) and their high mortality emphasizes the need for accessible perinatal care in Cambodia [7].

We are investigating whether pre-treatment with incomplete or sub-therapeutic antimicrobial regimens, especially in an unregulated private sector, contributes to low blood culture yield and high-levels of resistance in Cambodia. Resolution of conflicting demands for rational antibiotic prescribing to help prevent further emergence of antimicrobial resistance, and the urgent need for successful treatment of patients, would be aided by a network of sentinel microbiology laboratories throughout South-East Asia [3], [4]. Without such laboratories, efforts to treat severe infections in low and middle-income countries will flounder in the dark [39].

## Supporting Information

Figure S1 **Flowchart summarising methods for the analysis of cerebrospinal fluid (CSF) from children with suspected meningoencephalitis enrolled in the study.**
(PDF)

Figure S2 **Number of admissions with “acute serology” (see main text for definition) to DENV against period of admission (30-day intervals starting from study start date 12th October 2012).**
(PDF)

Figure S3 **Number of admissions with “acute/recent serology” (see main text for definition) to DENV against period of admission (30-day intervals starting from study start date 12th October 2012).**
(PDF)

Table S1 **Classification of DENV and JEV serology in samples.** ^a^ Dynamic rise of ≥2 Panbio units between acute and discharge samples. ^b^ Dynamic fall of ≤2 Panbio units between acute and discharge samples. ^c^ Dynamic rise or fall of ≤2 panbio units between acute and discharge samples. ^d^ Considered negative by manufacturer’s criteria.
(PDF)

Material S1 (DOC)
