# Correction: A Prospective Study of the Causes of Febrile Illness Requiring Hospitalization in Children in Cambodia

**DOI:** 10.1371/journal.pone.0119976

**Published:** 2015-03-26

**Authors:** The PLOS ONE Staff

The fifteenth author’s name is spelled incorrectly. The correct name is: Daniel H. Paris
