# Supplementary figures and images for: A Prospective Study of the Causes of Febrile Illness Requiring Hospitalization in Children in Cambodia
Source: PLoS One. 2013 Apr 9;8(4):e60634. doi: 10.1371/journal.pone.0060634 (PMC3621876; doi:10.1371/journal.pone.0060634)

**Figure S1.**

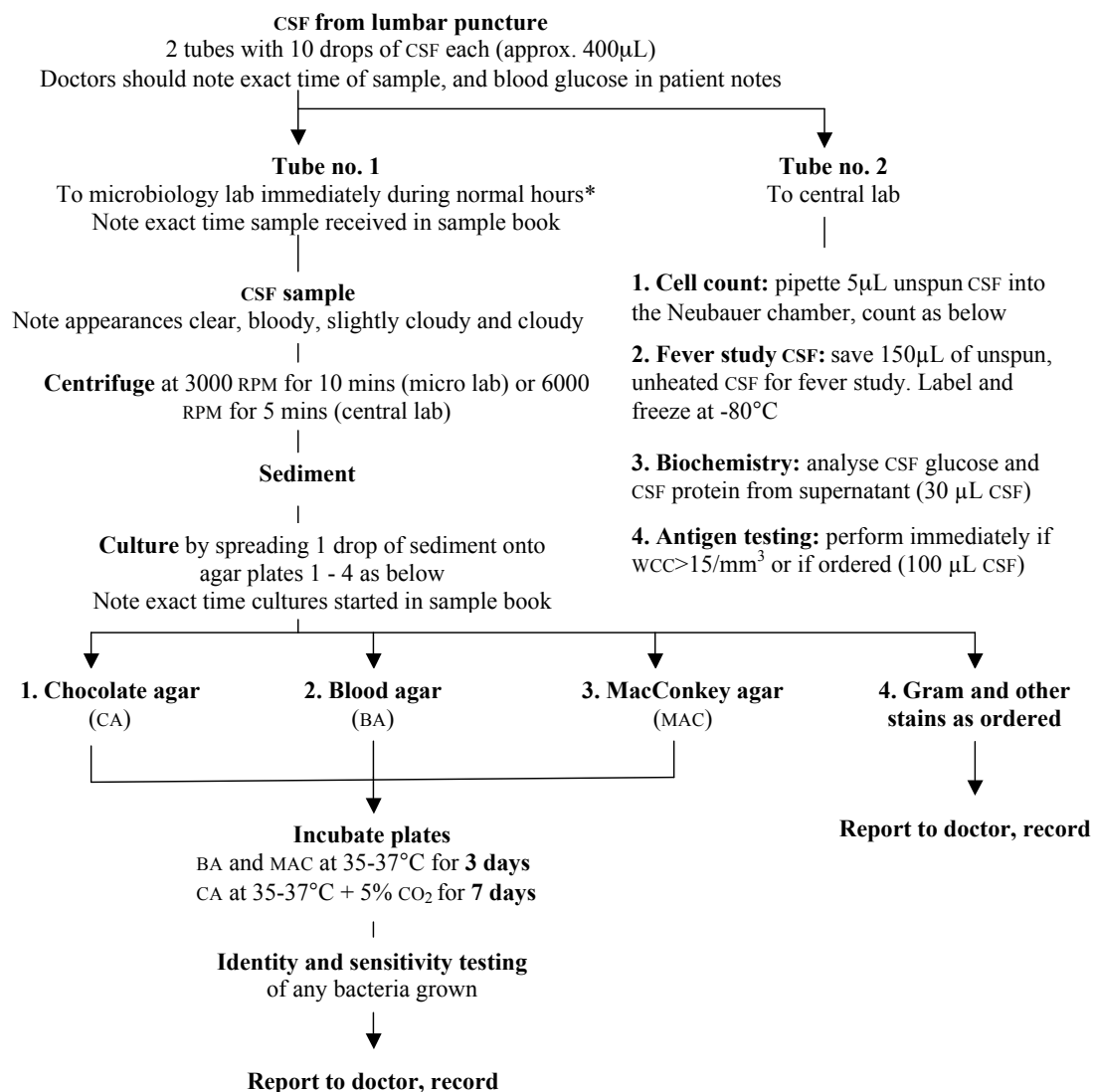

Supplement: Figure S1 — Flowchart summarising methods for the analysis of cerebrospinal fluid (CSF) from children with suspected meningoencephalitis enrolled in the study. (PDF) [file pone.0060634.s001.pdf]

Figure S2.

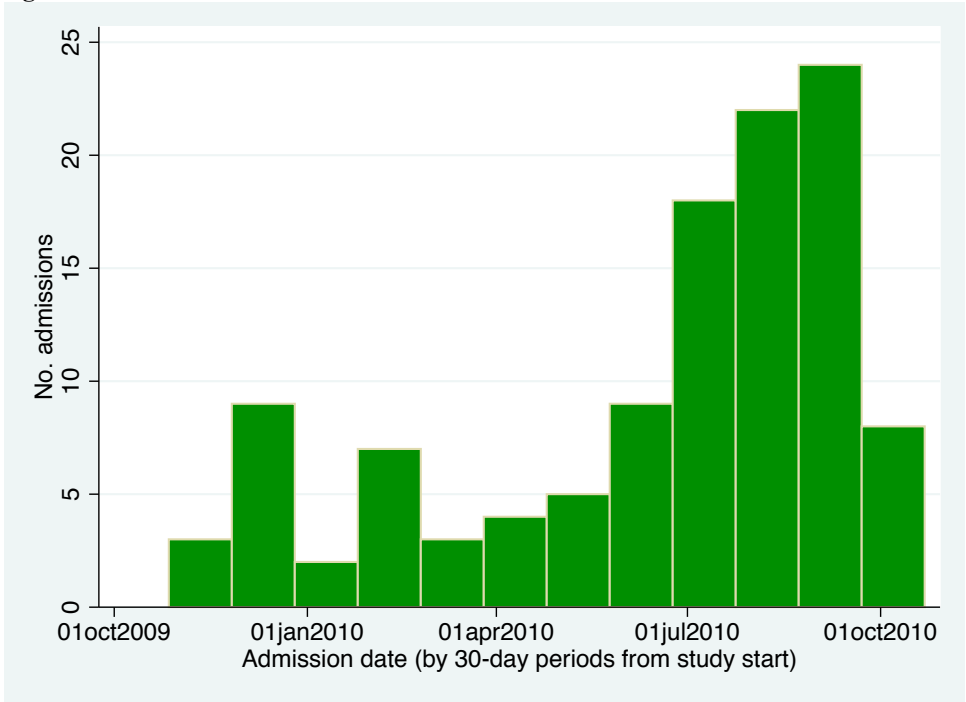

Supplement: Figure S2 — Number of admissions with “acute serology” (see main text for definition) to DENV against period of admission (30-day intervals starting from study start date 12th October 2012). (PDF) [file pone.0060634.s002.pdf]

Figure S3.

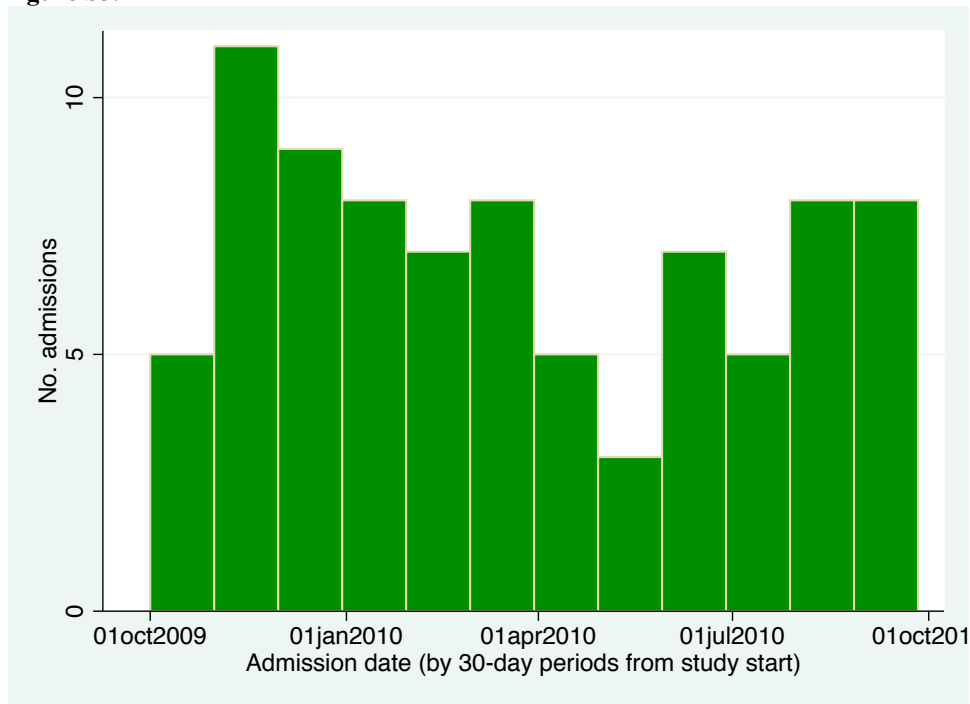

Supplement: Figure S3 — Number of admissions with “acute/recent serology” (see main text for definition) to DENV against period of admission (30-day intervals starting from study start date 12th October 2012). (PDF) [file pone.0060634.s003.pdf]
