# Supplementary material for: A Prospective Study of the Causes of Febrile Illness Requiring Hospitalization in Children in Cambodia
Source: PLoS One. 2013 Apr 9;8(4):e60634. doi: 10.1371/journal.pone.0060634 (PMC3621876; doi:10.1371/journal.pone.0060634)
Supplement: Table S1 — Classification of DENV and JEV serology in samples. a Dynamic rise of ≥2 Panbio units between acute and discharge samples. b Dynamic fall of ≤2 Panbio units between acute and discharge samples. c Dynamic rise or fall of ≤2 panbio units between acute and discharge samples. d Considered negative by manufacturer’s criteria. (PDF) [file pone.0060634.s004.pdf]

Table S1.

	Diagnosis	DENV NS1	Paired sera DENV IgM	JEV IgM	Single serum DENV IgM	JEV IgM	JEV/ DENV ratio
**DENV**	Acute DENV	Positive	Negative, static, falling	Rising[a], falling[b], static[c], negative[d]			<1
	Acute DENV	Negative, positive	Rising	Rising, falling, static, negative			<1
	Acute/recent DENV	Negative	Static	Static, negative			<1
	Acute/recent indeterminate flavivirus	Negative	Falling	Static, negative			<1
	Acute/recent indeterminate flavivirus	Negative	Rising	Negative, static, falling			>1
	Acute/recent DENV				Positive	Positive, negative	<1
**JEV**	Acute JEV	Negative	Negative, static, falling	Rising			>1
	Acute/recent JEV	Negative	Negative, static	Static			>1
	Acute/recent indeterminate flavivirus	Negative	Negative, static	Falling			>1
	Acute/recent indeterminate flavivirus	Negative	Negative, static, falling	Rising			<1
	Acute/recent JEV				Negative, positive	Positive	>1
**Mixed Dengue/ JEV**	Acute/recent indeterminate flavivirus	Negative	Falling	Falling			>1 or <1
