# Supplementary material for: A Prospective Study of the Causes of Febrile Illness Requiring Hospitalization in Children in Cambodia
Source: PLoS One. 2013 Apr 9;8(4):e60634. doi: 10.1371/journal.pone.0060634 (PMC3621876; doi:10.1371/journal.pone.0060634)
Supplement: Material S1 — (DOC) [file pone.0060634.s005.doc]

**SUPPORTING INFORMATION**

**METHODS**

**Bacterial culture from blood**

*Production of culture media*

Blood culture bottles: 37 g of brain heart infusion broth (Oxoid no. CM225, Oxoid, UK) was mixed with 0.25 g polyanatholesulfonate in 1 L of distilled water. 20 mL of the liquid was then distributed into each new, clean glass bottles manufactured for purpose (PN labs, Thailand). Each bottle was carefully sealed with rubber seals and aluminium tops with a cappers-hand-operated press, before being sterilised using an autoclave at 121 °C for 15 min. Blood culture bottles were produced on a fortnightly basis, kept for not longer than 3 months away from direct sunlight, and subject to rigorous quality checking.

Chocolate agar (modified Thayer Martin agar): 18 g GC agar base (CM0367, Oxoid) was mixed with 240 mL distilled water and brought to the boil gently, before being sterilised with an autoclave at 121 °C for 15 min. In addition 5 g of soluble haemoglobin powder (LP0053, Oxoid) was mixed with 250 mL distilled water and brought to the boil gently, before being sterilised with an autoclave at 121 °C for 15 min. The two solutions were then cooled to 50 °C in a water bath before both being mixed together and with 10 mL Vitox supplement (SR0090A, Oxoid) to the GC agar base. The haemoglobin solution was added aseptically to the GC agar base and mixed gently. The solution was poured into sterile Petri dishes.

Sheep blood agar: 39 g of Columbia blood agar base (CM0331, Oxoid) was suspended in 1 L distilled water and brought to the boil gently then autoclaved at 121°C for 15 minutes to sterilise. The media was cooled in a waterbath at 50 °C then 50 mL (5%) sterile citrated sheep blood was added aseptically then mixed gently. 18 mL was poured into each agar plate aseptically and each batch underwent quality control testing.

MacConkey agar: 52 g of MacConkey agar powdered medium medium (CM7, Oxoid) was dissolved in 1 L of distilled water, brought to the boil gently, then sterilised with an autoclave at 121 °C for 15 min. After cooling to 50 °C the solution was poured into sterile Petri dishes.

Ashdown agar: Ashdown agar was used for the selective isolation of *B. pseudomallei* only. The agar was produced with 10 g Tryptone soya broth casein digest medium (CM129, Oxoid), 15 g agar (LP0011, Oxoid), 40 mL glycerol (K26494594918, Merck, Germany), 5 mL crystal violet 0·1% (C0775, Sigma, Germany), 5 mL neutral red 1% (340564A, BDH, Netherlands), and 950 mL distilled water. The solution was sterilised 121°C for 15 min. After cooling to 50 °C, gentamicin was added to a concentration of 5 mg/L, before the solution was poured into sterile Petri dishes. Note: the crystal violet was incubated at 37 °C for two weeks before use.

Mueller-Hinton agar and Mueller-Hinton blood agar: Mueller-Hinton agar was used as a medium for antimicrobial susceptibility testing with Mueller-Hinton sheep blood agar used for fastidious organisms. 38 g of Mueller-Hinton powdered medium (CM337, Oxoid) was dissolved in 1 L of distilled water, and brought to the boil gently, before being sterilised with an autoclave at 121 °C for 15 minutes. After cooling to 50 °C the solution was poured into sterile Petri dishes with 5% citrated sheep blood mixed into the Mueller-Hinton agar prior to pouring aseptically for the Mueller-Hinton sheep blood agar.

*Culture and subculture methods including antibiotic sensitivities*

Standard methods were used for subculturing onto chocolate and blood agar at 24 h, 48 h and 7 days after start of aerobic incubation at 37 °C in the microbiology laboratory [1]. MacConkey agar and Ashdown agar were used where appropriate. Organisms were identified with standard methods and API test kits (bioMérieux, France) when appropriate [1]. We used disc diffusion methods [2], and Etests^TM^ (AB Biodisk, Sweden) following the CLSI guidelines as appropriate for antimicrobial susceptibility testing.

ESBL screening methods: Used for Gram-negative bacilli resistant to cefpodoxime on first-line antibiotic susceptibility testing. If ESBL positive on screening test, cefpodoxime, cefpodoxime with clavulanic acid, ceftazidime, ceftazidime with clavulanic acid, cefotaxime and cefotaxime with clavulanic acid; if the zone of inhibition between any of the antibiotic and the antibiotic plus clavulanic acid are difference more than 5 mm, the isolate was reported as an ESBL producing strain.

**Bacterial culture from CSF**

CSF samples were separated and analysed as described in **Figure S1**.

*Media production, culture and subculture methods including antibiotic sensitivities*

Media production, culture and subculture methods were as for isolates from blood culture (above).

**Serological assays**

*Dengue and JEV serology*

The Panbio Japanese Encephalitis Dengue IgM Combo ELISA was used (Panbio, Australia; Cat. # E-JED01C; Lot # 110061) [3]. Panbio Units were calculated by multiplying the index value (calculated by dividing the sample absorbance by the cut-off value (the average absorbance of the three calibrators) by 10.  The results were classed as negative for dengue and JEV if PanBio units were <9, equivocal if 9-11 and positive if >11. If both the dengue and JEV IgM results were positive, the JEV result was divided by the dengue result to give a ratio, with >1 indicating JEV infection and <1 indicating dengue infection. All ELISAs were repeated if the positive, negative or calibrator samples were out of range. All equivocal results were repeated.  If the repeat result was also equivocal the sample was reported as negative.  Dengue NS1 antigen ELISA (Standard Diagnostics, Korea; Lot # 224007) was used to detect dengue antigen [3]. To interpret the final patient results, the results of the Dengue NS1 antigen and the Dengue/JEV IgM ELISA were combined for patients that had paired serum collections and those that only had one serum collected (**Table S1**). Sample positive for anti-DENV IgM in CSF (one episode) or anti-JEV IgM in CSF (6 episodes) were counted as "acute serology" regardless of other serological titres from blood.

O tsutsugamushi *serology*

Karp and Gilliam antigens were used (kindly supplied by Dr. Allan Richards) for the ELISA, with a diagnostic cut off of 0·2 nett optical density at 450 nm against a background absorbance. This corresponds to a 1:200–1:400 indirect immunofluorescence assay (IFA) titre, which is considered positive in the region (SD Blacksell, unpublished data) [4,5]. Paired samples that demonstrated a ≥4-fold increase between the paired specimens were considered to have an "acute infection", pairs with insufficient rise between the samples were considered to have an "acute/recent infection". If only one sample was collected from a patient that was ≥1:400 in IFA that patient was considered to have a recent/acute infection.

Rickettsia typhi *serology*

The *R typhi*  (Wilmington strain) antigen (kindly supplied by Dr. Allan Richards) was used with a diagnostic cut off of 0·2 nett optical density at 450 nm, corresponding to a 1:400 IFA titre. All ELISA positive samples were titrated in a two-fold series from 1:100 to 25 600 in an IgM IFA using the method described by Blacksell et al [5] to determine the endpoint. Paired samples that demonstrated ≥4-fold increases between the paired specimens were considered to have an "acute infection". Serum pairs with <4-fold increase in titres between the samples were considered to have an "acute/recent infection". If only one sample was collected from a patient that was ≥1:400 in IFA that patient was considered to have an "acute/recent infection".

**Nucleic acid amplification tests (NAATs)**

DNA extraction from whole blood samples was performed with a commercial kit (QIAamp® DNA Mini Kit, QIAGEN, Germany) according to the manufacturer’s instructions with a prolonged incubation for 30 min at 56 °C. The DNA yield of an initial volume of 200 µL of full blood was eluted into 50 µL of distilled water to serve as the template for PCR assays. The DNA was quantified using a NanoDrop® spectrophotometer (Thermo Scientific).

*Leptospira* spp.

A probe-based real-time PCR was performed [6,7]. 5 µL of DNA extract was used in a 20 µL reaction volume using Platinum® Quantitative PCR SuperMix-UDG (Invitrogen^TM^, USA) kits. Each run contained duplicate low-positive standard dilutions over 4 orders of magnitude (1 to 1 x 10^3^ (GE/reaction) of *L interrogans* serovar Lai strain Lai. Runs where the 10 copies/µL standards failed were repeated. All positive controls (from blood from a patient with proven *Leptospira* spp infection) were positive. Samples were considered positive if they contained 1 copy/μL DNA or above. Visual inspection of the rPCR curves was consistent with true positive results at 1 copy/μL DNA.

*Orientia tsutsugamushi*

A probe-based real-time PCR was based on the 47 kDa gene and performed as previously described by Jiang et al [8]. 1 µL of DNA extract was used in a 25 µL reaction using Platinum® Quantitative PCR SuperMix-UDG (Invitrogen^TM^, USA) kits. Each run contained duplicate low-positive standard dilutions (using linearized pGEM plasmids), down to 1 copy/µL DNA. Runs where the 10 copies/µL DNA standards failed were repeated. Samples were considered positive if they contained 10 copies/μL or above (although, in practice, there were no samples with 1–9 copies/μL DNA).

*Rickettsia typhi*

This probe-based real-time PCR was based on the *ompB* gene of *R typhi* and performed as previously described by Henry et al [9]. 1 µL of DNA extract was used in a 25 µL reaction volume using Platinum® Quantitative PCR SuperMix-UDG (Invitrogen^TM^, USA) kits. Each plate was run with duplicate low-positive standard dilutions (linearized pGEM plasmids). Runs where the 10 copies/µL DNA standards were not detected were repeated. Samples were considered positive if they contained 10 copies/μL or above (28 samples had 1–9 copies/µL DNA, only 1 of which was also positive for anti-*R typhi* IgM).

*Respiratory viruses*

Polyester or nylon flocked throat and nasal swabs from patients with suspected respiratory infections were immediately placed in transport medium (universal viral transport medium for virus, Becton Dickinson, France; SAS catalogue # 220221) and stored at 5 °C. They were couriered by air freight to the Center for Disease Control, Ministry of Health laboratories in Phnom Penh where they were immediately stored at –70 °C. One aliquot (of three) was processed immediately for influenza viruses. The QIAGEN commercial kit (QIAamp® Viral RNA mini kit, QIAGEN, catalogue # 52906) was used to extract RNA from the nasal and throat swab medium according to the manufacturer’s instructions. All specimens were tested for influenza virus, type A, type B, and subtypes H1N1, H1N1pdm, H3N2 and H5N1, by real-time reverse-transcription polymerase chain reaction (rRT-PCR), as described in protocol of the Center for Disease Control and Prevention (CDC), Influenza Division, Atlanta, USA [10]; and by multiplex RT-PCR for parainfluenza (PIV)1, PIV2, PIV3 and human respiratory syncytial virus (RSV).

RNA was extracted from 140 µL of each sample, using a commercial reagent (QIAamp® viral RNA mini kit, QIAGEN); whenever possible the extracts were tested immediately after extraction. If this was not possible, they were divided into two aliquots and kept frozen at –80 ⁰C. Each aliquot was used only once to avoid the loss of viral genomic material during repetitive freezing and thawing.

The influenza virus primer and probe were designed by CDC, Influenza Division, including the virus positive control and human specimen extraction control (Catalogue # FluRUO 01, 02, 03 and, FluSW04). The rRT-PCR enzyme mastermix, using the Invitrogen commercial kit (Invitrogen SuperScript^TM^ III Platinum® One Step Quantitative RT-PCR System, catalogue # 11732-020), was used to determine the influenza virus concentration. The iQ5 real-time PCR (Bio-Rad, USA) was used for amplification and detection, programmed for reverse transcription of samples for 30 min at 50 °C, Taq inhibitor activation for 2 min at 95 °C and followed by 45 cycles of amplification for 15 s at 95 °C and for 30 s at 55 °C.

Multiplex RT-PCR was targeted to four respiratory viruses, RSV, PIV1, PIV2 and PIV3. An internal quality control was included to check the extraction step and the presence of inhibitor of RT-PCR assay. This control consisted of glyceraldehyde-3-phosphate dehydrogenase (GAPDH) gene, which is normally transcribed in nasal mucosae cells; this gene was amplified with specific primer [11]. Primers targeted specifically the heamagglutinin neuraminidase genes of PIV-1, PIV-2 [12], and PIV-3 [13], and the hypervariable region in the 5’-non-coding region of RSV [14]. Positive controls were included in each multiplex RT-PCR. These consisted of RNA extracted from virus infected cells culture (Diagnostic Hybrids, USA) and mixed together. As a negative control, water was used instead of nucleic acid. The multiplex RT-PCR was single combine RT-PCR amplification, performed using one-step RT-PCR kit (Qiagen® One Step RT-PCR Kit, QIAGEN). The reaction mixture contained 5 µL of 5x RT-PCR buffer (2.5 mM MgCl_2_), 0.4 mM dATP, dGTP, dCTP and dTTP, 0.5 µM of each of the 10 primers (eight primers for target viruses, and two for GAPDH control) and 1 µL of enzyme mix. A 2.5 µL aliquot of RNA extract was added to give a final volume of 25 µL. The cycling conditions for the two RT-PCRs were: an initial cycle at 50 ⁰C for 30 min and 94 ⁰C for 15 min; followed by 40 cycles at 94 ⁰C for 30s, 55 ⁰C for 30s and 72 ⁰C for 1 min and a final incubation at 72 ⁰C for 10 min [15].

**CSF Analysis**

Following isolation of nucleic acid from CSF specimens, four real-time polymerase chain reaction (PCR) protocols were used for detection of *S pneumoniae*, *H influenzae* type b, *N meningitidis*, and *S suis* [16,17]. Real-time (reverse transcription [RT]) PCRs were used to detect herpes simplex virus (HSV) 1 and 2 [18], varicella zoster virus (VZV) [19], enteroviruses (generic and enterovirus 71-specific) [20,21], and human parechoviruses (generic) [22]. All PCRs were internally controlled using equine arteritis virus for RNA virus detection or phocid herpes virus for DNA viruses and bacteria [18]. All oligonucleotides were ordered from Sigma-Proligo (Singapore).

**Leptospirosis culture**

1 mL whole blood samples were taken aseptically on enrolment to the study from children aged >60 days as described in Methods into sterile sodium heparinised tubes for culture of *Leptospira* spp. Samples for *Leptospira* spp. culture were stored securely in sterile tubes at room temperature in the Microbiology Laboratory at AHC until transported to the Mahidol-Oxford Tropical Medicine Research Unit, Bangkok, Thailand in batches approximately every two weeks for further culture as described below.

Culture of leptospires from blood was performed using 3 mL of *Leptospira* Medium Base Ellinghausen, McCullough, Johnson, and Harris (EMJH) medium (2.3 g/L, Difco, New Jersey, USA), *Leptospira* Enrichment EMJH (100 mL/L, Difco), and sodium pyruvate (10 mg/L, Merck) supplemented with 3% rabbit serum and 0.1% bacteriological agar (Agar No. 1, Oxoid, Hampshire, England) in 5 mL sterile plastic, flat based screw cap tubes (Sterilin, Barloworld Scientific Ltd, UK). Two sampling methods were used to isolate leptospires in the following order. (1) Whole blood: 100 µL of heparin blood was placed directly into EMJH. (2) Deposit from spun plasma: the remaining of heparin blood was centrifuged at 3,000 rpm for 10 min and left undisturbed for 30 min after which surface plasma was carefully removed and placed into 2 new tubes of equal volume which was centrifuged at 6,000 rpm for 3 min. The supernatant was removed to leave a 200 µL volume of deposit, which was re-suspended and placed, into 3 mL of semi-solid EMJH.

All cultures were incubated aerobically and light protected at room temperature (25-30 °C) for three months, and examined weekly for 4 weeks, then every 2 weeks for a further 2 months by placing one drop of culture onto a microscopic glass slide and observing by dark-field microscopy at 200x magnification. Positive cultures were referred to the WHO/FAO/OIE Collaborating Center for Reference & Research on Leptospirosis, Australia for identification using the cross agglutinin absorption test (CAAT).

**REFERENCES**

1. Murray PR, Baron EJ, Jorgensen JH, Pfaller MA, Tenover FC (2007) Manual of Clinical Microbiology (9^th^ Edition Revised). Washington DC, USA: American Society for Microbiology.

2. Clinical and Laboratory Standards Institute (2012) Performance standards for antimicrobial susceptibility testing. Supplement M100-S22. Wayne, PA, USA: Clinical and Laboratory Standards Institute.

3. Blacksell SD, Mammen MP, Thongpaseuth S, Gibbons RV, Jarman RG, et al. (2008) Evaluation of the Panbio dengue virus nonstructural 1 antigen detection and immunoglobulin M antibody enzyme-linked immunosorbent assays for the diagnosis of acute dengue infections in Laos. Diagn Microbiol Infect Dis 60(1): 43–9.

4. Blacksell SD, Bryant NJ, Paris DH, Doust JA, Sakoda Y, Day NP (2007) Scrub typhus serologic testing with the indirect immunofluorescence method as a diagnostic gold standard: A lack of consensus leads to a lot of confusion. Clin Infect Dis 44(3): 391­–401.

5. Blacksell SD, Jenjaroen K, Phetsouvanh R, Tanganuchitcharnchai A, Phouminh P, et al. (2010) Accuracy of rapid IgM-based immunochromatographic and immunoblot assays for diagnosis of acute scrub typhus and murine typhus infections in Laos. Am J Trop Med Hyg 83(2): 365–9.

6. Slack A, Symonds M, Dohnt M, Harris C, Brookes D, Smythe L (2007) Evaluation of a modified Taqman assay detecting pathogenic *Leptospira* spp against culture and *Leptospira*-specific IgM enzyme-linked immunosorbent assay in a clinical environment. Diagn Microbiol Infect Dis 57(4): 361–6.

7. Thaipadunpanit J, Chierakul W, Wuthiekanun V, Limmathurotsakul D, Amornchai P, et al. (2011) Diagnostic accuracy of real-time PCR assays targeting 16S rRNA and lipl32 genes for human leptospirosis in Thailand: A case-control study. PloS One 6(1): e16236.

8. Jiang J, Chan TC, Temenak JJ, Dasch GA, Ching WM, Richards AL (2004) Development of a quantitative real-time polymerase chain reaction assay specific for *Orientia tsutsugamushi*. Am J Trop Med Hyg 70(4): 351–6.

9. Henry KM, Jiang J, Rozmajzl PJ, Azad AF, Macaluso KR, Richards AL (2007) Development of quantitative real-time PCR assays to detect *Rickettsia typhi* and *Rickettsia felis*, the causative agents of murine typhus and flea-borne spotted fever. Mol Cell Probes 21(1): 17–23.

10. WHO Collaborating Centre for Influenza (2009) CDC rRT-PCR Protocol for Detection and Characterization of Influenza; version 2009 CDC Ref. # I-007-05. Geneva, Switzerland: World Health Organization.

11. Gueudin M, Vabret A, Petitjean J, Gouarin S, Brouard J, Freymuth F (2003) Quantification of respiratory syncytial virus RNA in nasal aspirates of children by real-time RT-PCR Assay. J Virol Methods 109: 39–45.

12. Echevarría JE, Erdman DD, Swierkosz EM, Holloway BP, Anderson LJ (1998) Simultaneous detection and identification of human parainfluenza virus 1, 2 and 3 from clinical samples by multiplex PCR. J Clin Microbiol 36: 1388–91.

13. Karron RA, Froelich JL, Bobo L, Belshe RB, Yolken RH (1994) Rapid detection of parainfluenza virus type 3 RNA in respiratory specimens: use of reverse transcription-PCR-enzyme immunoassay. J Clin Microbiol 32: 484–8.

14. Cane PR, Pringle CR (1991) Respiratory syncytial virus heterogeneity during an epidemic: analysis by limited nucleotide sequencing (SH gene) and restriction mapping (N gene). J Gen Virol 72: 349–357.

15. Bellau-Pujol S, Vabret A, Legrand L, Dina J, Gouarin S, et al. (2005) Development of three multiplex RT-PCR assays for the detection of 12 respiratory RNA viruses. J Virol Methods 126: 53–63.

16. Le VT, Phan TQ, Do QH, Nguyen BH, Lam QB, et al. (2010) Viral etiology of encephalitis in children in southern Vietnam: Results of a one-year prospective descriptive study. Plos Negl Trop Dis 4(10): e854.

17. Jerome KR, Huang ML, Wald A, Selke S, Corey L (2002) Quantitative stability of DNA after extended storage of clinical specimens as determined by real-time PCR. J Clin Microbiol 40(7): 2609–11.

18. van Doornum GJ, Guldemeester J, Osterhaus AD, Niesters HG (2003) Diagnosing herpesvirus infections by real-time amplification and rapid culture. J Clin Microbiol 41(2): 576–80.

19. de Jong MD, Weel JF, Schuurman T, Wertheim-van Dillen PM, Boom R (2000) Quantitation of varicella-zoster virus DNA in whole blood, plasma, and serum by PCR and electrochemiluminescence. J Clin Microbiol 38(7): 2568–73.

20. Beld M, Minnaar R, Weel J, Sol C, Damen M et al. (2004) Highly sensitive assay for detection of enterovirus in clinical specimens by reverse transcription-PCR with an armored RNA internal control. J Clin Microbiol 42(7): 3059–64.

21. Khanh TH, Sabanathan S, Thanh TT, Thoa LPK, Thuong TC, et al. (2012) A large outbreak of Enterovirus 71 associated hand, foot and mouth disease in southern Vietnam, September-November 2011. Emerg Infect Dis 18(12): 2002–5.

22. Benschop K, Molenkamp R, van der Ham A, Wolthers K, Beld M (2008) Rapid detection of human parechoviruses in clinical samples by real-time PCR. J Clin Virol 41(2): 69–74.

23. Wuthiekanun V, Chierakul W, Limmathurotsakul D, Smythe LD, Symonds ML, et al (2007) Optimization of culture of *Leptospira* from humans with leptospirosis. J Clin Microbiol 45(4): 1363–5.
